# A Survey of Course Leaders’ Perceptions of the Organizational Readiness to Respond to Gender-Based Violence at Swedish Civic Orientation for Refugees

**DOI:** 10.1177/10778012251379450

**Published:** 2025-09-22

**Authors:** Anna Pérez-Aronsson, Maissa Al-Adhami, Anna Sarkadi, Georgina Warner

**Affiliations:** 1 Child Health and Parenting (CHAP), Department of Public Health and Caring Science, 8097 Uppsala University, Uppsala, Sweden; 2 Centre for Women's Mental Health During the Reproductive Lifespan—WOMHER, 8097 Uppsala University, Uppsala, Sweden; 3 Equity and Health Policy, Department of Global Public Health, 27106 Karolinska Institutet, Stockholm, Sweden

**Keywords:** gender-based violence, intimate partner violence, domestic violence and abuse, sexual violence, refugees, help-seeking, Civic Orientation, organizational readiness

## Abstract

Gender-based violence (GBV) is a human rights issue with serious consequences. This cross-sectional study surveyed 108 Swedish Civic Orientation (CO) course leaders to assess organizational readiness to respond to GBV among newly arrived refugees. While most had received GBV training (78%) and rated their service's understanding as good (81%), fewer felt they had enough time (54%) or clear guidelines to act (27%). Open-ended responses highlighted needs for tailored materials, more training, stronger collaboration with support services, and dedicated time. Findings suggest CO course leaders are well-placed to detect GBV and link refugees to support, but further resources are needed.

## Background

Gender-based violence (GBV) is a human rights violation defined as acts and threats of physical, sexual, psychological, or financial harm conducted toward someone based on power imbalances and socially ascribed gender differences (Council of Europe, 2011). It can be perpetrated in public and private life, by family members as well as an unknown person; thus including different forms of violence such as domestic violence and abuse (DVA) and sexual violence (SV), which are the focus of this study. DVA and SV affect women across the world (Sardinha et al., 2022, 2024), and are associated with health problems such as chronic pain (Schou-Bredal et al., 2022), depression (Lohmann et al., 2024; Schou-Bredal et al., 2022) and posttraumatic stress disorder (Lohmann et al., 2024). GBV is understood to be rooted in power dynamics and gender norms; an interplay of personal, situational and sociocultural factors and intersecting systems of oppression can increase the risk of GBV and of facing negative consequences of violence, while concurrently creating barriers to support (Montesanti & Thurston, 2015; Reilly et al., 2022). Migration can amplify the risk of experiencing such socially vulnerable circumstances; and refugees have been reported to experience different forms of GBV across their migration trajectories, from premigration to resettlement, by a variety of perpetrators including armed forces, community leaders, police and border guards, asylum professionals, and family members (Pérez-Vázquez & Bonilla-Campos, 2023; Sullivan et al., 2021).

Multiple circumstances have been described as contributing to putting refugees at risk of GBV—including an increased incidence or exacerbation of DVA —and creating barriers to support. This include social isolation (Allen-Leap et al., 2023), financial difficulties (Wachter, Cook Heffron, Dalpe, & Spitz, 2021), immigration policies and fear of deportation (Canning, 2019), racism (Ahmad, 2019; Allen-Leap et al., 2023), stigma (Wachter, Cook Heffron, Dalpe, & Spitz, 2021), and concerns for having your children taken into custody (Allen-Leap et al., 2023). Studies on help-seeking for DVA in resettlement have highlighted barriers related to limited knowledge about available services (Wachter, Cook Heffron, Dalpe, & Spitz, 2021) and legal entitlements (Femi-Ajao et al., 2020). Further, language and interpretation can cause challenges in communication and trust building with service providers (Allen-Leap et al., 2023; Femi-Ajao et al., 2020; Wachter, Cook Heffron, Dalpe, & Spitz, 2021). Issues include lack of interpreters, concerns for confidentiality (Wachter, Cook Heffron, Dalpe, & Spitz, 2021) and varying quality of interpreters, with examples reported of interpreters withholding information in accounts of DVA (Femi-Ajao et al., 2020).

### Relational Aspects of Help-Seeking Processes

Theoretical models on seeking help for DVA highlight the relational aspects of help-seeking processes (Liang et al., 2005; Waller et al., 2023). Liang et al. (2005) draw on the cognitive theory of help-seeking in “stigmatizing” situations and describe a three-part process of defining the problem, deciding to seek help, and selecting a source of support. This process is not necessarily linear; for instance, the source of support chosen by the individual will inﬂuence how the problem is defined, and whether they will seek help again. Similarly, Waller et al. (2023) describe a help-seeking process that is mediated by the response of the support source. The initial phases of awareness, acknowledgement, and assessment are followed by a phase of “enough is enough,” after which the woman enlists help, escalates to trained providers, but then faces rejection and needs to persist despite barriers to support—this is termed the resolve phase, which is finally followed by restoration in which the woman can reclaim her sense of self. These theoretical models are supported by empirical evidence, which shows that perceived negative social interactions can constitute a barrier to help-seeking whereas perceived supportive responses can increase the likelihood of seeking help (Allen-Leap et al., 2023; Melgar Alcantud et al., 2021). Further, receiving negative social reactions to disclosure of interpersonal violence (including DVA and SV) has been shown to be associated with more severe symptoms of psychopathology; in particular, reactions involving attempts to control the person disclosing (Dworkin et al., 2019). Receiving positive social reactions, such as listening empathetically or providing tangible aid and information without forcing those exposed to engage with suggested services, has been found to be associated with better health outcomes (Dworkin et al., 2019; Sylaska & Edwards, 2014). Thus, considering how communities respond to disclosures of GBV is crucial for the health, well-being and access to support of those exposed to violence.

The concept of *provider trustworthiness* has been suggested to shift the focus from barriers that individuals must overcome to consider how services and systems can foster trust among individuals exposed to IPV (Kennedy et al., 2024). The concept includes three domains: *benevolence, fairness,* and *competence*. Kennedy et al. (2024) argue that the framework should be applied to a wide range of services, not only DVA support services, as personnel at community-based services, such as police stations, might also encounter individuals exposed to DVA and can be important for linking them onwards. Empirical evidence supports the importance of considering how a wide range of services and community members respond to DVA and SV. Not all women experiencing DVA and high levels of psychological distress seek help from social services or women's shelters (Dufort et al., 2013), and many turn to family, friends, community groups, and coworkers for support (Melgar Alcantud et al., 2021; Sylaska & Edwards, 2014). However, migration often entails the scattering of established social networks, and women have described how separation from family and friends has meant losing important sources of support for DVA (Wachter, Cook Heffron, & Dalpe, 2021). Further, friends who are resettling themselves can feel uninformed and ill-equipped to help (Wachter, Cook Heffron, Dalpe, & Spitz, 2021). Broader research on how refugees rebuild social support in resettlement shows that personnel at community sites and services regularly accessed by refugees can be important sources of social support for refugees in resettlement, particularly personnel who share the same language (Wachter et al., 2022). Aligning with this, studies have found that community-based service providers such as hairstylists (Kim, 2021) or caseworkers at resettlement agencies (Wachter, Cook Heffron, Dalpe, & Spitz, 2021) might receive disclosures of DVA from refugees visiting their service, and provide them with information or help in contacting support services.

### The Swedish Civic Orientation Course as a Potential Arena for Identifying Support Needs

A previous formative research process, consisting of workshops with women with experiences of migration and GBV and various service providers, pointed to the need to explore Swedish Civic Orientation (CO) courses as a community-based service with potential to detect GBV and connect those in need with support (Pérez-Aronsson et al., 2024). CO is a course that the Swedish municipalities are obliged to offer to newly settled refugees who hold a temporary or permanent residence permit (SFS 2010:1138). The course is part of a 2-year Introduction Program that includes a language course and job-seeking and vocational training activities aimed at increasing refugees’ knowledge about society and facilitating employment and social integration. The Introduction Program is offered to people between 20 and 66 years, who have recently been granted a residence permit in Sweden as a refugee, a person in need of subsidiary protection, or under the Temporary Protection Directive, or are next-of-kin to people granted residence permit related to those conditions (SFS nr: 2017:820). The CO course covers four areas; practical information about everyday life, how society is organized, human rights, and Swedish democratic values and rights and obligations of the individual. In addition to the municipalities, CO can also be arranged by private actors, and delivered on site or online. It is most commonly offered in the participants’ native language or in another language they master well; however, delivery in easy Swedish or via interpreter also occurs (Länsstyrelsen Värmland, 2023). It is provided over a minimum of 100 hr by course leaders referred to as civic communicators, who speak both Swedish and the language spoken by their course participants and often have own experiences of migrating to Sweden (Svensson et al., 2021b). Currently, there are no formal educational requirements to be employed as a civic communicator, meaning their background professions and training might vary (Länsstyrelsen Värmland, 2023; Svensson et al., 2021b). According to a survey conducted by the Swedish County Administrative Boards (2023), regional government agencies that, among other policy implementation duties, are tasked by the Swedish government to conduct yearly reports on CO, there is a need to increase civic communicators’ competence, in particular, their ability to manage conversations on sensitive topics. *Organizational readiness* for change is key for enacting such a shift in personnel competence (Weiner, 2009). Organizational readiness is a complex construct, encompassing several factors. A fundamental component is the value that is placed on the change and that personnel feel capable of achieving the change, including having the necessary resources. The broader organizational context is also considered essential, including organizational policies and procedures. Exploring civic communicators’ perspectives on the organizational readiness to respond to DVA and SV at CO services can contribute to knowledge useful for enhancing provider trustworthiness, which in turn could decrease the risks of unsupportive responses to disclosures and strengthen the potential of CO to link to support services.

### Aim

To explore civic communicators’ perceptions of the organizational readiness to respond to domestic violence and abuse and sexual violence at Swedish Civic Orientation for newly arrived refugees.

### Public Involvement

This study was conceptualized through a series of codesign workshops involving women with lived experience of migration and some form of GBV, alongside personnel from relevant services, including civic communicators and CO managers (Pérez-Aronsson et al., 2024). Through these workshops, for which the aim was to codesign a service model for refugee women in Sweden who have experienced GBV, the public contributors and service personnel brought attention to CO services as an arena to acquire societal information, with the potential to lower thresholds for seeking support services, but recognized that this might require additional resources and training for civic communicators. The public contributors played a foundational role in shaping the research question addressed in this article. Their contributions during the codesign process were instrumental in identifying the service context and research focus, without which this study would not have been pursued. However, they did not take an active role in subsequent stages of the research, including data collection, analysis, or dissemination, but rather asked to be kept informed of progress. This selective involvement reflects the practical constraints of contributor capacity and the broader structure of the research program, which comprises multiple substudies stemming from the original codesigned service model. In this instance, the contributors, while recognizing the value of this particular study, chose to direct their efforts toward other areas of the program where their input was deemed most impactful. This selective engagement underscores the notion that meaningful public involvement does not require uniform participation across all phases of the research cycle. Rather, involvement should be flexible and responsive to both the contributors’ expertise and availability, as well as the needs of the wider research program.

## Method

### Sample and Data Collection

A cross-sectional study design was used, in which data were collected through a web-based survey spread to civic communicators across Sweden between September 2023 and May 2024. The aim was to reach the total population of civic communicators in Sweden. The exact number is not known, since there is no national record of the professional group and the turnover of staff is high (Länsstyrelsen Värmland, 2023). To disseminate the survey, e-mails were sent to the CO coordinators at the County Administrative Boards (*N* = 21). The e-mail contained information about the survey that the CO coordinators were asked to spread onwards to all civic communicators in their region, which included a QR code and link to the web-survey. In some municipalities, contact was also made directly with managers at CO services. Two reminders were sent, with a few weeks in between. Confirmation was received that the survey had been sent out in 19 of the 21 regions in Sweden, reaching at least 174 civic communicators. The municipalities in one region had CO courses delivered through services in another region, instead of arranging themselves, and one region did not respond. It is possible that the survey link was spread more widely. The data were collected using REDcap, a secure web application specifically designed to support the online collection of research data.

Since this study used an anonymous web-survey and no personal data were collected, it did not fall within the Swedish ethical legislation according to which ethical clearance is not required for anonymous research (SFS 2003:460). The research was planned with the professionals’ comfort and confidentiality in mind; for example, they did not have to state which municipality they work in, the survey only made enquiries about their work duties; not personal experiences, and it did not introduce an unfamiliar topic since GBV is included in the existing CO curriculum.

### Measures

The web-survey contained a 13-item scale exploring the civic communicators’ view on the organizational readiness to respond to SV and DVA (hereafter summarized as GBV), complemented by four questions on current GBV practices, a set of demographic questions, and an open-ended question asking what the civic communicators thought their service should do to help course participants exposed to GBV (see Appendix 1). The 13-item scale on organizational readiness was derived from the 12-item H-REMIS scale, which has been previously developed from the PREMIS tool and the Consolidated Framework for Implementation Research constructs to assess the organizational readiness for intimate partner violence response among housing providers (Kaur et al., 2022). The original PREMIS tool (Short et al., 2006) was developed to assess physicians’ readiness to respond to IPV (Short et al., 2006). The briefer H-REMIS scale has demonstrated acceptable internal consistency reliability (Cronbach's α = .748) (Kaur et al., 2022).

For this study, the H-REMIS scale was translated from English to Swedish with slight adaptions to the context of CO services, such as changing the word “*clients*” to “*course participants*.” The translation was independently cross-checked by three native Swedish speakers fluent in English (one researcher, one NGO staff, and one Swedish teacher). The translated scale was presented to a group of CO coordinators and managers, who were asked to consider if the questions made sense from their perspective and the CO-context. Their reflections led to the addition of the four complementary questions exploring current practices relevant to GBV, namely, on holding classes on violence (see Appendix 1, part 1). While the responses to these questions were handled separately in the analyses and not as part of the organizational readiness scale, the phrasing of the questions was modeled on the H-REMIS scale for consistency across the survey. As a usability test, three Swedish-speaking PhD students—with backgrounds in social work, nursing and political sciences—answered the full survey in REDcap and provided feedback. This led to the identification of perceived unclarities in the phrasing of one of the items of the organizational readiness scale, which, in response was divided into two (Appendix 1, part 2, Q11 and Q12). The final adapted and translated 13-item CO version of the organizational readiness scale had a Cronbach’s alpha of .801, indicating good internal consistency. The four subscales in the scale demonstrated varying internal consistency: Cronbach’s alpha .701 for GBV perceptions, .656 for GBV policies, .795 for Staff training, and .643 for Staff capacity and resources.

### Analysis

Descriptive analyses were used to summarize demographic characteristics, current GBV practices, organizational readiness factors and the overall organizational readiness score. The reported percentages were calculated based on the number of responses for each item, which varied across the survey. The statistical analyses were conducted using Excel and R Studio—version 2023.03.0, build 386 (RStudio Team, 2020).

Qualitative content analysis (Graneheim & Lundman, 2004) was used to analyze responses to the open-ended question. The first author (APA) read all responses to obtain a sense of the whole. Most responses could be handled as a single meaning unit, but some needed to be divided into multiple meaning units. As the responses were already short and concentrated, condensation was not necessary. Instead, the next step of analysis consisted of labeling the meaning units with codes that were kept close to the text. These codes were compared with consideration to similarities and differences and grouped into five subcategories. An overarching category was created to capture the essence of all five sub-categories. The second author (MA) read all responses and crosschecked the codes, subcategories and overall category created by the first author (APA). Then, the authors discussed the analysis, leading to a refinement of the name of the overarching category.

### Researcher Positionality

The authors are affiliated with a research group situated within a public health department and use a social determinants of health framework and a health equity perspective as points of departure in their work. APA is a PhD student writing her thesis on the community's role in recovery from GBV and forced migration. MA is a researcher working on projects related to migration and GBV. Her doctoral studies focused on health promotion in the early postmigration phase, including evaluating the delivery of health communication and mental health courses at the Swedish Civic Orientation. AS is a professor in social medicine who has been involved in various research projects related to migration and health. GW is an associate professor in child health and welfare, currently leading a participatory research project on psychosocial support for experiences of GBV and forced migration. The authors have previous experience in mixed-method and qualitative research studies, including the analysis methods employed in this study. In addition to their experiences of conducting research related to migration and GBV, the authors have personal connections to the topic of migration: AS and GW migrated to Sweden from other European countries as adults. MA came to Sweden with her family as a young child, as a refugee from the Middle East. APA has a parent who came to Sweden as a refugee from South America.

## Results

**Figure 1.**
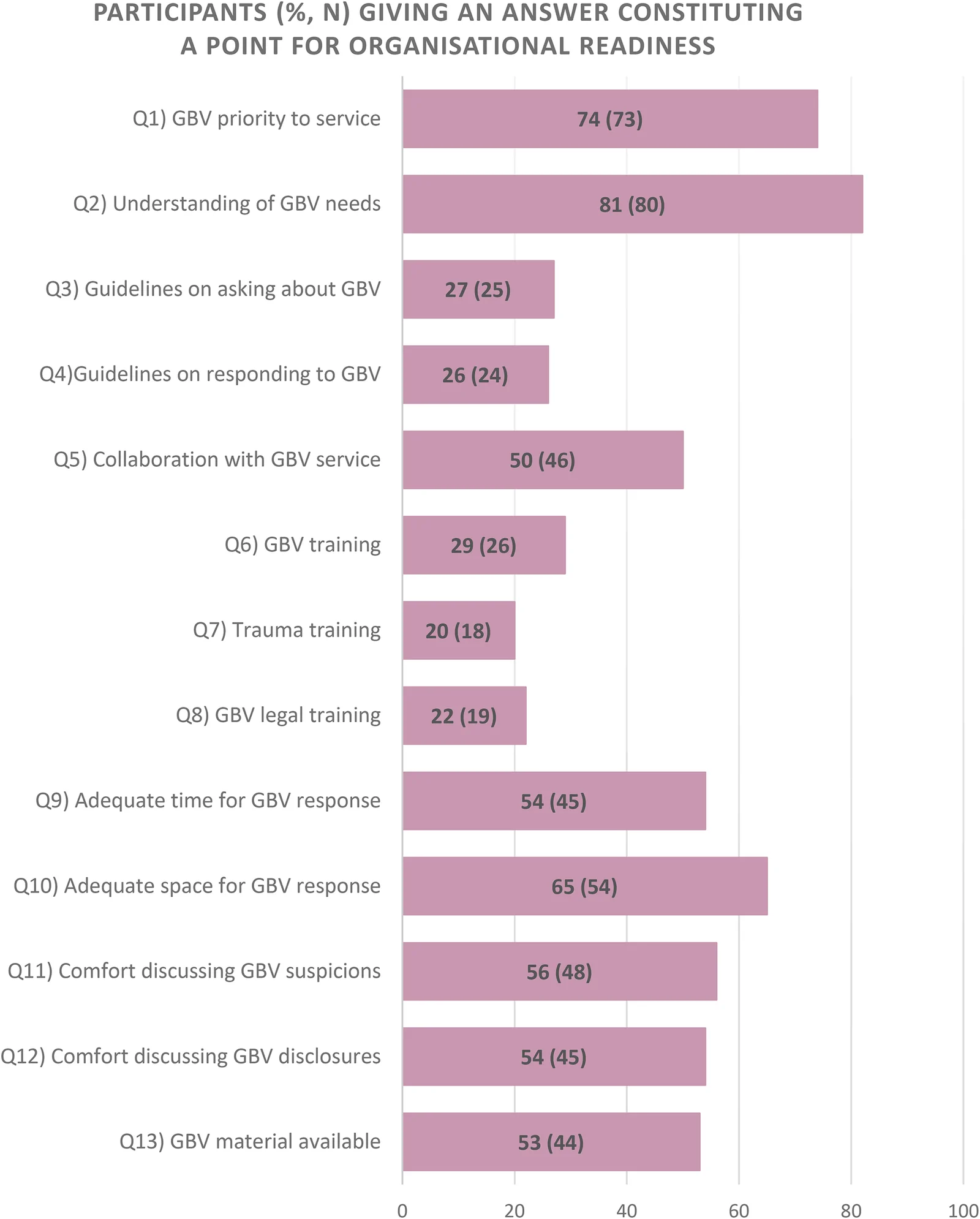
Amount of participants (%) giving an answer constituting a point on each item of the organizational readiness scale.

A total of 108 (62% of confirmed recipients) participants responded, at least partially, to the survey. The fewest responses for an individual item on the organizational readiness scale was *n* = 83. Answers to the open-ended question were provided by *n* = 30 participants. The participants’ demographic characteristics are presented in Figure 1. The sample included participants working across Sweden, in small, medium, and large municipalities. The majority (58%, *n* = 43) of the participants had a university-level education, had received specific CO-training (65%, *n* = 48) and training on GBV (78%, *n* = 58). Slightly more identified as women (48%, *n* = 34) than men (41%, *n* = 29) and the mean age was 51 years, with a mean of 7.4 years working as a civic communicator. Almost half (43%, *n* = 32) reported having had concerns, or being made aware, that a course participant was exposed to GBV. Analysis of current GBV practices showed that the majority (80%, *n* = 86) regarded delivering lessons on GBV as a high or fairly high priority to their service, but only 37% (*n* = 40) said that all the staff at their service delivered such lessons (see Table 1).

**Table 1.** Participant Demographics and Current GBV^a^ Practices.

Item	Participants % (*N*)^b^
Participant demographic	
** **Gender identity	Women 48 (34) men 41 (29) do not want to state 11 (8)
Educational level	
Secondary school	12 (9)
University <3 years	30 (22)
University >3 years	58 (43)
CO^c^-training	
Yes	65 (48)
No	35 (26)
GBV^a^ training	
Yes	78 (58)
No	22 (16)
Municipality size	
Small (<40,000 inh.)	39 (29)
Medium (40,000 < 200,000 inh.)	32 (23)
Big (at least 200,000 inh.)	28 (21)
Location	
North Sweden	16 (12)
Mid Sweden	41 (30)
South Sweden	42 (31)
Suspected or been made aware that a course participant was exposed to GBV^a^	
Yes	43 (32)
No	57 (42)
Variable (*N*)	Mean years (min, max)
Age (63)	51 (16, 70)
Years delivering CO^c^ (73)	74 (1, 11)
Participant ratings of current CO^c^ GBV^a^ practices	
Importance GBV^a^ lessons	
** **Fairly/very high	80 (86)
GBV^a^ lessons delivered	
** **Yes, all staff	37 (40)
Allow dialog at GBV^a^ lessons	
Yes, to a great extent	55 (59)
Comfort holding GBV^a^ lessons	
Fairly/very comfortable	63 (68)

a GBV (gender-based violence) is here used to summarize *sexual violence* and *domestic violence* *and abuse* (in Swedish, “violence in near relationships”), the terms used throughout in the Swedish version of the survey.

b Total *N* varies due to partial responses.

c Civic Orientation for newly-arrived refugees.

### Ratings of Organizational Readiness

The participants’ ratings of the organizational readiness to respond to GBV at their service are displayed in Figure 1. While the majority said GBV was a high or fairly high priority for their service (74%, *n* = 73) and rated service understanding of GBV needs as good or very good (81%, *n* = 80), ratings were lower for the questions on training and guidelines on GBV. Less than a third were aware of all staff at their service receiving training on detecting and responding to GBV (29%, *n* = 26), and a fifth said all staff had training on trauma (20%, *n* = 18). Around a quarter said their service had widely used guidelines on asking about (27%, *n* = 25) and responding to GBV (26%, *n* = 24). More than half felt they had enough time to respond to GBV (54%, *n* = 45) and an adequate private space to do so (65%, *n* = 54), and rated the staff comfort level when discussing GBV with course participants as fairly or very comfortable when being concerned that someone was experiencing GBV (56%, *n* = 48) and when someone disclosed GBV (54%, *n* = 45). Half knew of cooperation with GBV support services (50%, *n* = 46). The mean readiness for GBV response score was 6.1, with a standard deviation of 3.2.

### Being a Link Between the Exposed and Services Requires Organizational Support

The qualitative analysis of the open-ended question on what the participants thought their services should do to support individuals exposed to GBV resulted in the creation of one main category “*Being a link between the exposed and services requires organizational support*.” This category aimed to capture the overarching finding identified through analysis, namely, that the participants regarded CO services to have the potential to support individuals exposed to GBV and link them onwards to help—but that this required organizational support for personnel. The main category was formed from five subcategories, all detailing different supportive structures and resources that were described as important for supporting CO course participants experiencing GBV and handling GBV in a sensitive way in lectures. Namely, supporting course participants experiencing GBV and linking them onwards to help was expressed to require* GBV guidelines*, *tailored GBV material, time*, *training on detecting and responding to GBV* and *collaborations with GBV support services such as social services*. It varied whether these structures were perceived to be in place; some civic communicators gave examples of existing guidelines and collaborations that helped them support course participants exposed to GBV whereas others expressed wishing that such structures existed.

To really cooperate with the social service's DVA unit and be a link between the exposed and the authority. To gain more knowledge and authorization to be able to support the exposed. (Woman, medium-sized municipality in South Sweden, with 1–5 years CO teaching experience)

Regarding tailored GBV material, various suggestions were given concerning what was needed to handle the topic of GBV within CO courses in a safe, respectful way and avoid causing feelings of shame, including adjustment of GBV lecture material and teaching formats, such as holding lessons in groups separated by gender, offering resources on safety strategies, and arranging support groups. More *time* was asked for—in class, to be able to cover the topic of GBV carefully, as well as more nonteaching time to enable GBV responses and referrals of individuals in need.

It's important to adapt the language to the target group and be patient in explaining and normalising the concept, i.e., to be able to talk about this without it feeling like a taboo subject or causing shame for the individual. (Woman, small-sized municipality in South Sweden, with 1–5 years CO teaching experience)

Offer courses and resources that help participants to develop self-awareness and strategies to avoid dangerous situations. Create support groups where participants can share their experiences and support each other in a safe and respectful environment. (Man, small-sized municipality in North Sweden, >5 years CO teaching experience)

Aligning with the perceived potential that GBV might be detected at CO and referrals to support services could be made, it was expressed that migrants other than those entitled to take part in CO as part of the Introduction Program might be experiencing GBV and thus might benefit from receiving the information offered at the CO courses. Moreover, it was raised that civic communicators’ existing relationship with the course participants could be a potential barrier in cases of GBV, as both victim and perpetrator might be attending the same course. Collaboration with GBV services was suggested as a way to handle this issue.

The [CO] service could collaborate with GBV experts. When violence within a relationship is detected, it can be challenging for [CO] staff to be impartial as they have already had time to build some sort of relationship with the people concerned, for example, two course participants in the same [CO] course who have a relationship with each other. In such cases, it can be better for someone else to jump in. Instead, the [CO] service can train staff on behaviour, and to be able to interpret signs that a course participant might need help as there are those who suffer in silence. (Woman, medium-sized municipality in North Sweden, >5 years’ CO teaching experience)

Importantly, this last quote indicates a perceived need for training in recognizing signs of distress in course participants, as some individuals might not directly express their exposure to violence or need of support.

## Discussion

The findings of this study indicate that there is potential for CO services to be an arena where refugees with experiences of GBV could be identified, receive information, and be connected with support services. Almost half of the civic communicators participating in this study said they had suspected or been alerted to GBV exposure among course participants, thus supporting the hypothesis suggested in previous formative research (Pérez-Aronsson et al., 2024). The majority viewed responding to and supporting course participants exposed to GBV as important for their service and rated their service understanding of the needs of individuals exposed to GBV as fairly or very high. Drawing on the concept of provider trustworthiness, this indicates a high benevolence at CO services toward individuals exposed to GBV (Kennedy et al., 2024). However, the findings of this study also pointed to opportunities for enhancing the organizational readiness to respond to GBV at CO services.

Less than a quarter of the respondents in the present study reported that all staff received training on GBV and trauma. Competence is a key domain of provider trustworthiness (Kennedy et al., 2024) and these findings highlight training as a clear development area for the CO context. Training has previously been found to increase civic communicators’ confidence in leading discussions on topics perceived as sensitive such as mental health (Al-Adhami et al., 2023) and, once again looking to the healthcare context, there is a plethora of evidence on DVA training. A systematic review highlighted that training appears to improve personnel knowledge of DVA, their attitudes toward individuals exposed to violence, and their perceived readiness to respond; however, it is unclear whether the training influences actual response behaviors and the likelihood of referral to support services (Kalra et al., 2021). An important difference between healthcare and the CO context is that civic communicators are expected to manage group discussions on sensitive topics; it is stated in the law that the education should allow for dialog (SFS 2010:1138). Only around half (55%, n 59) of the civic communicators in this study said dialog was allowed to a great extent at GBV lessons, and the qualitative findings indicate that civic communicators perceive additional resources might be needed for GBV to be discussed in a safe environment, such as creating smaller groups separated by gender and adapting teaching material to be able to discuss the concept of GBV without creating feelings of shame or taboo for individuals. These findings align with previous research demonstrating that norms of shame and taboo can be experienced to be reinforced when discussing sensitive topics such as sexual and reproductive health and rights information at mixed gender CO courses (Svensson et al., 2017), moreover, these findings suggest that civic communicators might harbor concerns that allowing GBV discussions in their classroom could be hurtful for individuals experiencing violence, which should be further explored. Another distinction between healthcare and the CO context worth noting is that civic communicators could be viewed as cultural mediators, often sharing linguistic or cultural backgrounds with participants (Svensson et al., 2021a). Yet, aside from one mention of a need for greater cultural competence, the participants in the current study did not reflect on how shared cultural backgrounds influenced their readiness to address GBV, suggesting this may be an important area for further exploration.

Around half of the civic communicators participating in the study said staff at their service were comfortable discussing GBV suspicions and disclosures and had adequate space and time to do so. A synthesis of qualitative evidence from the healthcare context has highlighted that how personnel talk about DVA and having adequate time for the conversation is of great importance (Korab-Chandler et al., 2022), demonstrating a potential for improving CO services’ readiness to respond sensitively to individuals experiencing GBV. Interestingly, a greater proportion of civic communicators in this study reported that staff at their service were comfortable in discussing suspicions or disclosures of GBV, compared to the proportion that reported that all staff at their service received formal training on the topic. While this may superficially suggest that training is unnecessary, it may instead reflect a lack of awareness regarding the complexity and sensitivity of GBV-related interactions. Untrained professionals may overestimate preparedness, while training could lead to a more nuanced understanding of appropriate responses. Thus, this finding could signal unrecognized gaps in knowledge rather than genuine readiness to respond.

The association between trauma training and comfort in discussing GBV should not be overlooked. Training in trauma-informed care can help equip personnel by increasing their understanding of the needs of people exposed to violence, particularly related to the fundamental need for safety in the context of the caregiving relationship (Sperlich et al., 2021). The qualitative findings of this study indicate a further potential benefit in raising civic communicators’ trauma awareness; a desire was expressed for training to recognize signs of distress in course participants who do not explicitly express their suffering. The silence that often surrounds trauma and experiences of violence has been previously highlighted, for instance, in a previous qualitative study women resettling as refugees in the United States described keeping silent about DVA for various reasons, such as norms that being a women entailed suffering, or as a strategy to keep the family safe (Wachter, Cook Heffron, Dalpe, & Spitz, 2021). A study analyzing demographic and health survey data from a large sample of women in sub-Saharan countries demonstrated that 61% of women with experiences of DVA had not sought help (Dickson et al., 2023). Further, women with experiences of DVA have reported feeling invisible in contact with Swedish healthcare and wishing that personnel had asked them about abuse (Pratt-Eriksson et al., 2014). In addition to the evidence suggesting that training is associated with healthcare personnel's perceived readiness to respond to GBV (Kalra et al., 2021), the lack of clear service guidelines on how to handle GBV disclosures has been highlighted as a source of discomfort for personnel (Anderzén Carlsson et al., 2021). The qualitative findings in this study showed that civic communicators viewed guidelines as helpful for responding to GBV. Given that less than a third of the civic communicators in this study stated that their service had guidelines on asking about and responding to GBV, the development and implementation of guidelines could help improve CO services’ readiness to respond to individuals exposed to GBV in a trustworthy manner.

Previous research (Kim, 2021; Wachter, Cook Heffron, Dalpe, & Spitz, 2021, Wachter, Cook Heffron, & Dalpe 2021) has suggested that community-based providers with established relationships with refugees might be well-placed to detect GBV, given the importance that trust-building and getting to know each other play for help-seeking. While the findings of this study largely align with these previous studies—around half of the participating civic communicators said they had experiences detecting GBV in course participants—it was also highlighted that established relationships can pose a challenge for providers, if they are familiar with and meet the perpetrator too. A need to reach a broader group of migrants who might currently be underserved was indicated in the open-ended responses. However, this needs to be reconciled with the challenge of having both victims and perpetrators of GBV attend the same sessions. While gender-specific classes have been raised as a solution, there is a risk that this approach might reinforce binary, heteronormative or stereotypical views of gender and violence, and could also create logistical complications for the service. On account of the known high turnover of civic communicators (Länsstyrelsen Värmland, 2023), it is also important to consider the brevity and frequency of GBV training to be embedded into CO services. Frequent and brief training could enable sufficient repetition for new personnel to be trained promptly, with the added benefit of refreshing the competence of longer-term personnel, much like cardiopulmonary resuscitation training for healthcare personnel.

The results showed a high standard deviation in overall readiness scores, which could suggest differential possibilities for refugees to receive supportive responses and referrals to services through CO courses depending on where they attend the course or who their course leader is. This aligns with the qualitative findings of the study, which revealed that some respondents felt able to help those in need thanks to structures such as collaborations, whereas others expressed that they needed more training, collaborations, material, etc. Such differences in organizational readiness suggest that there might be room for improvement in the fairness domain of provider trustworthiness (Kennedy et al., 2024). This finding is, however, not very surprising given the decentralized nature of Sweden in which municipalities operate with a great level of independence. Even with an internationally strong policy on violence against women, Sweden suffers from implementation issues when translating the policy into concrete actions (Nyhlén & Giritli Nygren, 2019). This aligns with the low number of respondents who reported knowing of a local guideline on how to respond to GBV. The geographical location of CO sites could also be instrumental, as research exploring the implementation of GBV policy actions in the Swedish school system highlighted the influence of limited resources in rural locations, with a lack of prioritization of GBV affecting budget allocation (Nyhlén & Giritli Nygren, 2019).

A major limitation of this study is the risk of response bias. The total number of civic communicators in Sweden is not known, and there is no national directory to contact them. For this reason, the web-survey was distributed in an unrestricted nonlisted format, which carries a known risk to data quality (Callegaro et al., 2015). It was confirmed that 174 civic communicators received the survey; however, it is possible that the survey was spread more widely. It is also possible that those civic communicators who chose to participate in the study were those who found the topic of GBV important and relevant, which could lead to skewed results. Further, this study used only self-reported data, meaning social desirability could influence the responses. In addition, the data was collected in Swedish and while it is a requisite for civic communicators to speak Swedish, most do not have it as their native language, and their Swedish proficiency could vary. While the survey was based on an existing instrument and demonstrated a good level of internal reliability, it is the first time it has been used which brings uncertainty regarding validity. Moreover, there is no established benchmark regarding what constitutes a good level of organizational readiness. Therefore, the results need to be interpreted with caution and conclusions cannot be drawn regarding the organizational readiness to respond to GBV at CO services overall. Despite these limitations, the study served the purpose of pointing to development opportunities within the CO services. The findings on the heterogeneity of readiness scores and the low ratings on receiving GBV training and having GBV guidelines indicate actions that could be taken to strengthen the readiness to respond. That the sample included participants from across Sweden, in municipalities of different sizes, and with different amounts of CO teaching experience, is a strength in terms of representability.

To conclude, the findings from this study add to the literature suggesting that community-based services could be important arenas for detecting refugees in need of support for GBV and connecting them to relevant support services. The personnel participating in this study expressed that responding to GBV was an important priority for which they want training, resources and dedicated time. Based on the findings, several key actions are recommended. Clear and actionable guidelines should be developed to support CO course leaders in identifying and responding to GBV. Additional time and resources are needed within CO programs to ensure leaders can effectively address these issues. Ongoing, tailored training in GBV and trauma should be provided, along with the development of appropriate educational materials. Finally, stronger collaboration with specialized support services is essential to ensure timely and effective referrals.

## Appendix 1

**Table:**

Part 1. Current GBV^a^ practices^b^
How important is it that Civic Orientation contains classes on GBV^a^? Very low priority Somewhat low priority Neither high nor low priority Somewhat high priority Very high priority Does the staff at your service deliver lectures on GBV^a^? No Yes, some staff Yes, all staff Unsure When lectures about violence are delivered, does the staff leave room for dialog, discussion, and reflection? No Yes, to some extent Yes, to a large extent Unsure How comfortable are staff with delivering lectures about GBV^a^? Very uncomfortable Somewhat uncomfortable Neither comfortable nor uncomfortable Somewhat comfortable Very comfortable
Part 2. Organizational readiness^c^
*GBV^a^ perceptions*
How important is GBV^a^ response for your service? Very low priority Somewhat low priority Neither high nor low priority Somewhat high priority^+^ Very high priority^+^ How well do you think your service understands GBV^a^ survivors’ **needs?** Not well at all Not very well Neither well nor not well Well^+^ Very well^+^
*GBV^a^ guidelines*
3. Does your service have written guidelines in place for asking about GBV^a^? No Yes, not used Yes, used to some extent Yes, widely used^+^ Unsure 4. Does your service have written guidelines in place for GBV^a^ response if a course participant discloses GBV^a^ exposure? No Yes, not used Yes, used to some extent Yes, widely used^+^ Unsure 5. Does your service collaborate with an organization or service providing support to GBV^a^ survivors? Yes^+^ No
*Staff training*
6. Do staff receive training on identifying and responding to GBV^a^? No Yes, some staff Yes, all staff^+^ 7. Do staff receive trauma-focused training? No Yes, some staff Yes, all staff^+^ 8. Do staff receive training on legislation concerning GBV^a^? No Yes, some staff Yes, all staff^+^
*Staff capacity and resources*
9. Do staff have enough time to respond to course participants who have been exposed to GBV^a^? Yes^+^ No 10. Is there adequate private space to provide support to course participants exposed to GBV^a^ and refer them onwards? Yes^+^ No 11. How comfortable are staff with discussing GBV^a^ with course participants when there is a suspicion about GBV^a^ exposure? Very uncomfortable Somewhat uncomfortable Neither comfortable nor uncomfortable Somewhat comfortable^+^ Very comfortable^+^ 12. How comfortable are staff with discussing GBV^a^ with course participants who disclose their experiences? Very uncomfortable Somewhat uncomfortable Neither comfortable nor uncomfortable Somewhat comfortable^+^ Very comfortable^+^ 13. Are there material on violence exposure (like brochures, telephone numbers to support lines, etc.) at your service facility? No Yes, but not particularly visible Yes, visible, but not used Yes, visible and used^+^
Part 3. Demographics
Age Drop down menu ranging from 15 to >75 years Gender identity Woman Man Nonbinary Other Do not want to disclose Municipality Want to state the municipality's name Write the municipality's name Prefer not to state the municipality's name State municipality size (small <40,000 inh, medium between 40,000 and < 200,000 inh, or big at least 200,000 inh) State geographical location (North, Mid or South Sweden) What is your highest level of education? Elementary school Secondary school or equivalent University or equivalent < 3 years University or equivalent > 3 years For how long have you taught at civic orientation for newly arrived? Drop down menu from <1 year to >10 years Have you attended any education in civic and health communication such as MILSA^d^? Yes No Have you attended any education in GBV? Yes On how many occasions? Drop down menu from 1 to >5 No Have you experienced that there have been suspicions that a course participant is being exposed to violence, or that you have been made aware that a course participant is being exposed to GBV? Yes If you have any comments about that please write those here No
Part 4. Open-ended question
If you could decide, what would your service do to help course participants who have been exposed to GBV^a^?

a GBV is here used to summarize *sexual violence* and *domestic violence and abuse* (in Swedish, “*violence in near relationships”)*, the terms used throughout in the Swedish version of the survey.

b Items developed for this study based on the H-REMIS survey (Kaur et al., 2022).

c Items from the H-REMIS survey (Kaur et al., 2022), slightly adapted to the Civic Orientation context.

d MILSA is a research-based support and development platform focusing on promoting health and integration of newly arrived refugees.

+ Response constituted a point for organizational readiness on this survey item.
